# Predictive factors for outcome in HER2-low breast cancer patients after neoadjuvant chemotherapy

**DOI:** 10.3389/fonc.2025.1459444

**Published:** 2025-03-05

**Authors:** Yingbo Shao, Huijuan Guan, Zhifen Luo, Yang Yu, Yaning He, Qi Chen, Chaojun Liu, Fangyuan Zhu, Hui Liu

**Affiliations:** ^1^ Department of Breast Oncology, Henan Provincial People’s Hospital, Zhengzhou University People’s Hospital, Zhengzhou, Henan, China; ^2^ Department of Breast Oncology, Henan Provincial People’s Hospital, Henan University People’s Hospital, Zhengzhou, Henan, China; ^3^ Department of Pathology, Henan Provincial People’s Hospital, Zhengzhou University People’s Hospital, Zhengzhou, Henan, China; ^4^ Department of Pathology, Henan Provincial People’s Hospital, Henan University People’s Hospital, Zhengzhou, Henan, China; ^5^ Department of Medical Oncology, Henan Provincial People’s Hospital, Zhengzhou University People’s Hospital, Zhengzhou, Henan, China; ^6^ Department of Medical Oncology, Henan Provincial People’s Hospital, Henan University People’s Hospital, Zhengzhou, Henan, China

**Keywords:** breast cancer, neoadjuvant chemotherapy, HER2-low expression, pathological complete response, prognosis

## Abstract

**Objective:**

The present study aimed to evaluate the predictive factors that predict outcomes of HER2-low breast cancer patients who did not achieve pathological complete response(pCR) after neoadjuvant chemotherapy (NAC).

**Methods:**

This study included patients with HER2-low breast cancer who received NAC from January 2017 to December 2020. Analysis of the clinicopathological features, NAC response and outcome of the patients were retrospectively analyzed. Univariate and multivariable Cox analysis were used to determine factors that predict outcomes of HER2-low breast cancer patients who did not exhibit pCR.

**Results:**

293 Asian patients were included. The proportion of patients with hormone receptor (HR) positive and triple negative breast cancer (TNBC) among HER2-low patients was 75.8% and 24.2%, respectively. The pCR rate of HR positive cases was significantly lower than TNBC (27.5% vs. 53.5%, P=0.000). The patients who obtained pCR after NAC showed better disease-free survival(DFS) (5-year DFS 93.9% vs. 83.1%, p=0.039). For patients not achieving pCR, multivariable analysis showed that Miller/Payne (MP) grading system (hazard ratio: 0.094; 95% CI: 0.037-0.238; p=0.000) and HR status (hazard ratio: 2.561; 95% CI: 1.100-5.966; p=0.029) were significant independent predictors for DFS. Additionally, The MP grading system was also an independent predictor of overall survival (OS) (hazard ratio: 0.071; 95% CI: 0.019-0.260; p=0.000).

**Conclusions:**

The results of our study show that pathological assessment following NAC offers valuable insights into the survival outcome of HER2-low breast cancer. According to these findings, responses to NAC should be considered when choosing systemic treatment for patients with HER2-low breast cancer.

## Introduction

On the basis of differences in gene expression patterns, breast cancer can be divided into four clinical subtypes that have significant implications for treatment and prognosis: luminal A, luminal B, human epidermal growth factor receptor 2 (HER2) overexpression, and basal-like subtypes (1, 2). Twenty to twenty-five percent of these cases are HER2 positive breast cancers. The HER2 oncogene is a member of the ErbB tumor gene family, and it plays a crucial role in the biological behavior of breast cancer. The development of anti-HER2 targeted therapies, including trastuzumab, lapatinib, pertuzumab, and trastuzumab emtansine (T-DM1), has markedly improved the treatment outcomes of HER2-positive breast cancer patients (3–7). In 2018, the American Society of Clinical Oncology (ASCO) and the College of American Pathologists (CAP) clinical practice guideline proposed that patients whose immunohistochemistry (IHC) results show 0/1+ or IHC 2+ with negative *in situ* hybridization (ISH) should be diagnosed as having HER2-negative breast cancer, and those with IHC 3+ or IHC 2+ and ISH positive results should be diagnosed as having HER2-positive breast cancer (8).

With the advent of novel antibody-drug conjugate (ADC) drugs, the concept of HER2-low breast cancer has been proposed (9). HER2-low breast cancer refers to cancers with HER2 IHC staining results of 1+ or 2+ and negative ISH, results, and this kind of cancers accounts for 45% to 55% of breast cancer cases (10). HER2-low breast cancer is predominantly classified as HR positive/HER2-negative or TNBC. However, HER2-low and HER2-zero breast cancer differ not only in terms of HER2 protein expression level, but also in terms of estrogen receptor (ER) status, primary tumor volume, lymph node involvement, pathological complete response (pCR) rate after neoadjuvant chemotherapy (NAC), and disease-free survival (DFS) (11, 12). Furthermore, there is a notable difference in OS between HER2-low and HER2-zero breast cancer patients (13, 14). Although HER2-low breast cancer is not yet considered an independent molecular subtype, this does not affect the use of HER2-low breast cancer as a therapeutic target or exploration of the biological behavior of HER2-low breast cancer.

At present, neoadjuvant therapy has emerged as a crucial component of the systemic treatment of breast cancer (15, 16). Guided by the efficacy of neoadjuvant therapy, it is possible to identify patients with suboptimal clinical response, thereby allowing for the optimization of treatment protocols or the enhancement of adjuvant therapy, which may lead to improved prognosis in patients with breast cancer. Previous clinical trials on NAC have established that HER2-low breast cancer had a significantly lower pCR rate than HER2-zero patients (17–19). Further analysis demonstrated that among HR positive breast cancer patients, HER2-low patients typically presents a lower incidence of grade III tumors, a lower Ki-67 proliferation index, and fewer TP53 mutations compared with HER2-zero patients. Currently, few clinical studies have focused on prognostic factors specific to HER2-low breast cancer after NAC. The present study aimed to evaluate factors that predict outcomes in individuals with HER2-low breast cancer who did not achieve pCR after NAC.

## Materials and methods

### Study population

In this retrospective analysis, we screened women who were diagnosed with HER2-low breast cancer at Henan Provincial People’s Hospital (Zhengzhou, China) between January 2017 and December 2020. Patient participation in the analysis was contingent upon meeting the following criteria: 1) invasive breast cancer with histological confirmation; 2) HER2-low status as determined by IHC or fluorescence *in situ* hybridization (FISH); 3) received NAC; and 4) underwent subsequent breast surgery following NAC. The primary exclusion criteria were as follows: 1) lack of surgical intervention following NAC, which precluded any assessment of treatment efficacy on the basis of pathological evaluation; and 2) lack of available follow-up data.

### Pathological and clinical features

Clinical and pathological data were collected for each patient, including information on therapy, HER2 status, Ki-67 levels, nodal involvement, age at tumor diagnosis, histological type, clinical tumor size, clinical stage, and HR status. The clinical stage was determined using the seventh edition of the tumor−node−metastasis (TNM) staging system established by the American Joint Committee on Cancer (AJCC. A Ki-67 threshold of 30% was selected on the basis of several considerations. First, according to the 2024 guidelines of the Chinese Society of Clinical Oncology (CSCO), a Ki-67 index greater than or equal to 30% is classified as high. Additionally, the International Ki-67 Breast Cancer Working Group (IKWG) noted out that when the Ki-67 index is ≥30%, this index is reliable for the evaluation of prognosis. Furthermore, the major of patients included in this analysis presented with locally advanced breast cancer, with a limited number exhibiting a Ki-67 index less than 20%. IHC was utilized to assess progesterone receptor (PR) and ER, with a cut-off value of ≥1%. Positive ER and/or PR scores were considered to indicate a HR-positive status, whereas negative ER and PR scores were considered to indicate a HR-negative status. The HER2 status was evaluated according to the criteria set forth by the American Society of Clinical Oncology/College of American Pathologists (ASCO/CAP), with HER2-low was described as IHC 1+, or IHC 2+ and negative FISH results. HER2 expression data was retrieved from medical records, and reviewed by a pathologist. Currently, there is no global consensus regarding the age threshold for defining young breast cancer patients, with some studies setting this threshold at 35 years of age and some studies setting it at 40 years of age. In this study, we used 35 years old as the threshold for patients with young breast cancer.

### NAC and assessment of efficacy

NAC was administered to all subjects who were included in this study. The chemotherapy regimens utilized included anthracycline and taxane-based protocols, taxane combined with platinum regimens, and other regimens that aligned with established guidelines. After every two cycles of treatment, all the patients underwent imaging examinations, including MRI and ultrasounds, to assess clinical efficacy of the treatment. The vast majority of these patients received surgical intervention upon completion of all the NAC cycles. However, a small number may have undergone surgery prior to completing the full chemotherapy regimen due to chemical toxicity and other factors. The pathological evaluation criterion for pathological complete response (pCR) following NAC was defined as ypTis/0ypN0, indicating the absence of invasive cancer in the breast (regardless of ductal carcinoma *in situ*) and axillary lymph nodes.

For patients who did not achieve pCR, the HER2 status of residual disease was determined. The evolution rate means the overall rate of HER2 discordance from primary breast cancer to residual breast cancer, including HER2-low transitioned to HER2-zero (HER2 loss) and HER2-low transitioned to HER2-positive (HER2 gain).

The MP grading system was also used to evaluate the pathological response to NAC. Grade 1: no change or some change in individual malignant cells but no reduction in overall cellularity; Grade 2: a minor loss of tumor cells but overall cellularity remaining high; accounting for up to 30% loss; Grade 3: an estimated reduction in tumor cells between 30% and 90%; Grade 4: a marked disappearance of tumor cells such that only small clusters or widely dispersed individual cells remain; more than 90% loss of tumor cells; Grade 5: no malignant cells identifiable in sections from the site of the tumor, but ductal carcinoma *in situ* (DCIS) may be present (20).

### Statistical analysis

The clinical characteristics of the groups were compared using descriptive statistics. Fisher’s exact test or Pearson’s chi-squared test were used to compare the differences between the groups. The deadline for a follow-up was set to December 31, 2023. The median follow-up duration of the study patients was 49 months. From the date of surgery to the date of a distant relapse, locoregional relapse, death, or the last follow-up, was defined as disease free survival (DFS). From the date of surgery to the date to the patient’s death or the last follow-up, was defined as overall survival (OS). The Kaplan-Meier method was used to estimate the patient survival curves, and the log-rank test was used to compare them. Univariate and multivariable Cox analysis were used to identify characteristics predictive of survival result in patients without pCR, including age, cT, cN, clinical stage, HR, pre Ki-67, surgery, pT, pN, pathological stage, HER2, post Ki-67 and MP. Software from SPSS 22.0(SPSS Inc., IL, US) was used for all statistical descriptive analyses. P<0.05 was considered significant.

## Results

### Clinicopathological characteristics of the study population

In this investigation, 293 patients with HER2-low breast cancer who received NAC were retrospectively screened. **Table 1** presents the characteristics of the patients and their treatments. The proportion of patients with pure invasive breast cancer of no particular type (IBC-NST) in the whole population was 82.3%. The majority of patients included in this analysis had locally advanced breast cancer, the percentages of T3-4 and N2-3 patients were 30.0% and 41.3%, respectively. Among these patients, clinical stage II was observed in 137 individuals (46.8%), whereas stage III was observed in 156 individuals (53.2%). In total, 71 patients were diagnosed with TNBC, whereas HR-positive breast cancer was present in 222 patients (75.8%). A total of 75.8% of the population exhibited a Ki-67 percentage exceeding 30%. In this study, anthracycline- and taxane-based NAC therapy was administered to 96.6% of the patients. Following NAC, mastectomy was the most prevalent surgical procedure, the remaining 18.8% of patients underwent immediate breast reconstruction (IBR) or breast conserving surgery.

**Table 1:** Patient and treatment characteristics on tumor biopsy.

Characteristic	Total n=293	HR positive n=222	TNBC n=71	*P*
Age				0.817
≤ 35	69(23.5)	53(23.9)	16(22.5)	
> 35	224(76.5)	169(76.1)	55(77.5)	
Histology type				0.830
IBC-NST	241(82.3)	182(82.0)	59(83.1)	
Other	52(17.7)	40(18.0)	12(16.9)	
cT				0.618
T1-2	205(70.0)	157(70.7)	48(67.7)	
T3-4	88(30.0)	65(29.3)	23(32.4)	
cN				0.851
N0-1	172(58.7)	131(59.0)	41(57.7)	
N2-3	121(41.3)	91(41.0)	30(42.3)	
Clinical stage				**0.025**
II	137(46.8)	112(59.5)	25(35.2)	
III	156(53.2)	110(40.5)	46(64.8)	
HR				–
Positive	222(75.8)	222(100.0)	0(0)	
Negative	71(24.2)	0(0)	71(100.0)	
Ki-67				**0.000**
≤ 30%	71(24.2)	67(30.2)	4(5.6)	
> 30%	222(75.8)	155(69.8)	67(94.4)	
Chemotherapy regimen				**0.049**
Anthracycline + taxane	283(96.6)	217(97.7)	66(93.0)	
Platinum	6(2.0)	2(0.9)	4(5.6)	
Others	4(1.4)	3(1.4)	1(1.4)	
Surgery				0.221
Mastectomy	238(81.2)	180(81.1)	58(81.7)	
Breast conserving	40(13.7)	33(14.9)	7(9.9)	
IBR	15(5.1)	9(4.1)	6(8.5)	
pCR				**0.000**
Yes	99(33.8)	61(27.5)	38(53.5)	
No	194(66.2)	161(72.5)	33(46.5)	

IBC-NST, invasive breast cancer, no special type; HR, hormone receptor; IBR, immediate breast reconstruction. Bold value: there are statistical differences.

The biological activity associated with HER2-low breast cancer is significantly influenced by its HR status. The percentage of clinical stage III breast cancer in TNBC cases was considerably greater than that in their in HR positive counterparts (64.8% vs. 40.5%, P=0.025). Concurrently, there was a significant increase in the percentage of TNBC with Ki-67 > 30% compared with HR positive breast cancer (94.4% vs. 69.8%, P=0.000). A total of 5.6% of TNBC patients received platinum-based treatment. Other patient demographics and therapeutic characteristics were similar between TNBC patients and HR-positive breast cancer.

### Pathological response after NAC

**Figure 1:**
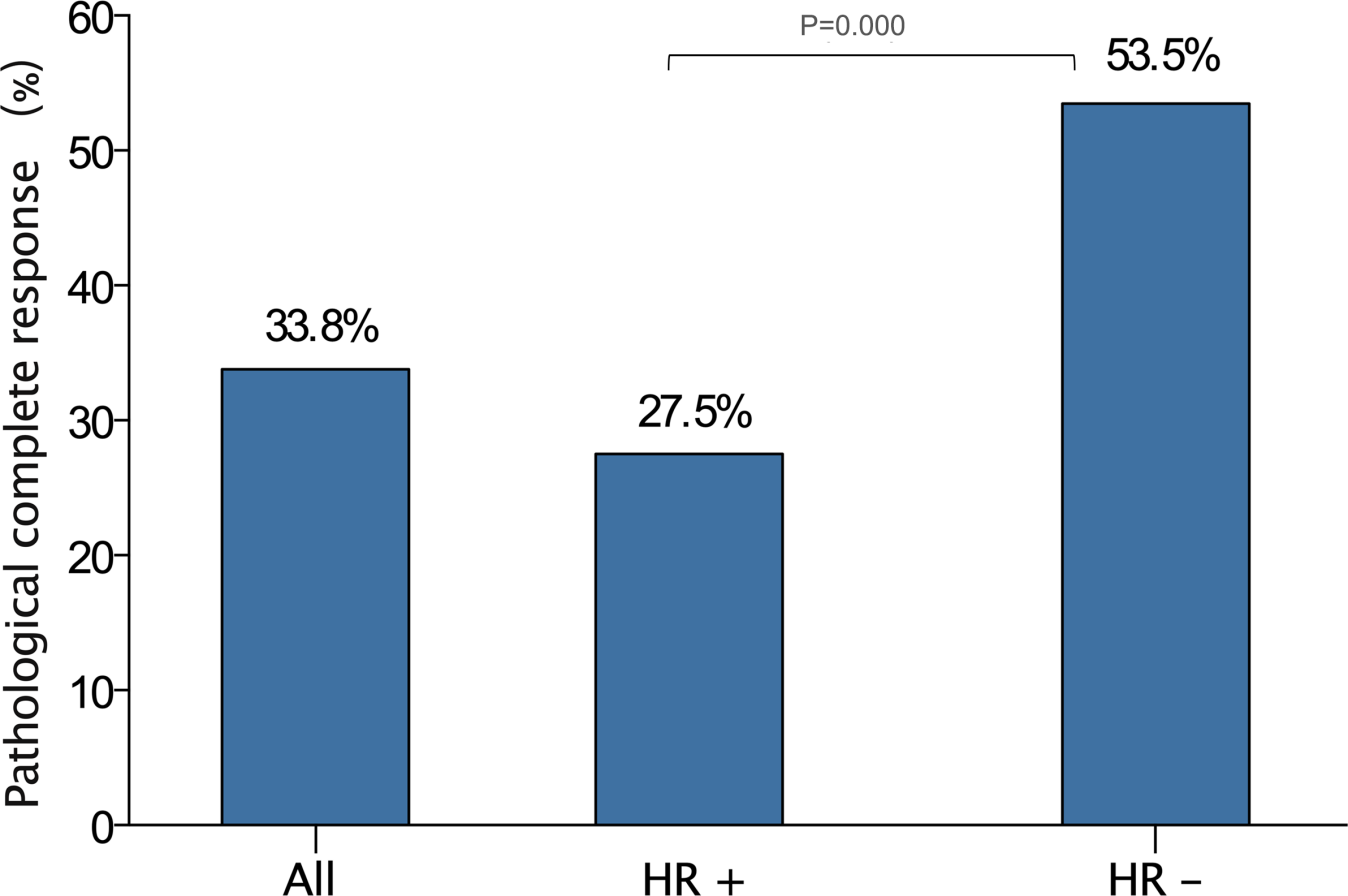
The pCR rate in the entire population, HR positive patients and TNBC.

Ninety-nine patients in the total population attained pCR, yielding a 33.8% pCR rate. Pathological response after NAC are summarized in **Table 2**. According to the MP grading system, 10 (3.4%) patients had a grade 1 response, 57 (19.5%) patients had a grade 2 response, 80 (27.3%) patients had a grade 3 response, 42 (14.3%) patients had a grade 4 response, and 104 (35.5%) patients had a grade 5 response. The pCR rate in TNBC was significantly higher than in HR positive breast cancer patients (53.5% vs. 27.5%, P=0.000, **Figure 1**). With respect to the MP grading system, the proportion of MP grade 1 and grade 2 tumors in TNBC was significantly lower than in HR positive breast cancer (12.7% vs. 26.1%, P=0.001). In this study, HER2 status was compared between residual disease after NAC and tumor biopsy specimen in patients who did not achieve pCR. Compared with HR positive patients, patients with TNBC exhibited high instability after NAC. The evolution rate means the overall rate of HER2 discordance from primary breast cancer to residual breast cancer. In patients with TNBC and HR positive disease, the evolution rate of HER2-low disease following NAC was 21.1% and 14.9%, respectively.

**Table 2:** Tumor characteristic on breast surgical tissue of non-pCR patients.

Characteristic	Total n=194	HR positive n=161	TNBC n=33	*P*
MP				**0.001**
1	10(5.2)	8(5.0)	2(6.1)	
2	57(29.4)	50(31.1)	7(21.2)	
3	80(41.2)	67(41.6)	13(39.4)	
4	42(21.6)	33(20.5)	9(27.3)	
pT				0.053
T0-2	180(92.8)	152(94.4)	28(84.8)	
T3-4	14(7.2)	9(5.6)	5(15.2)	
pN				**0.044**
N0-1	123(63.4)	97(60.2)	26(78.8)	
N2-3	71(36.6)	64(39.8)	7(21.2)	
Pathological stage				**0.000**
I	44(22.7)	24(14.9)	20(60.6)	
II	76(39.2)	70(43.5)	6(18.2)	
III	74(38.1)	67(41.6)	7(21.2)	
HER2				**0.010**
Stable	146(75.3)	128(79.5)	18(54.5)	
Loss	36(18.6)	25(15.5)	11(33.3)	
Gain	12(6.2)	8(5.0)	4(12.1)	
Ki-67				**0.000**
≤ 30%	139(71.6)	125(77.6)	14(42.4)	
> 30%	55(28.4)	36(22.4)	19(57.6)	

NAC, Neoadjuvant chemotherapy; pCR, pathological complete response; MP, Miller/Payne grading system. Bold value: there are statistical differences.

Compared with HR positive patients, patients diagnosed with TNBC were more likely to achieve pCR, and the proportion of MP1-2 was also lower.

### Follow-up and survival analysis

**Figure 2:**
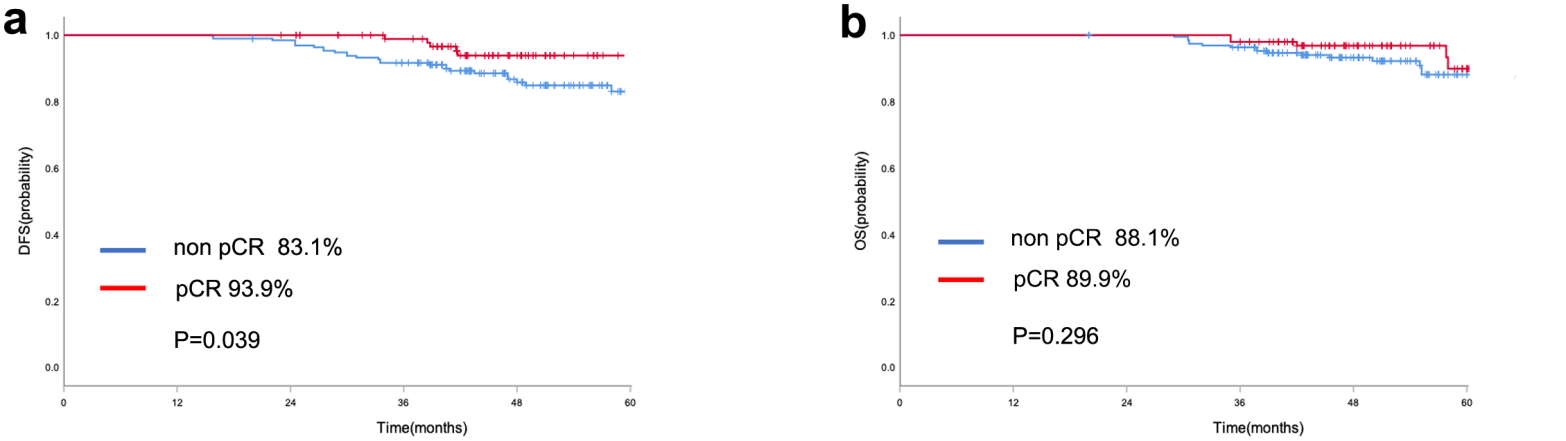
Kaplan-Meier curve of DFS **(a)** and OS **(b)** in the entire cohort with pCR and non-pCR.

As shown in this study, the patients who achieved pCR had a more favorable prognosis. Specifically, the 5-year DFS rate for patients who achieved pCR was considerably greater than that for those who did not achieve pCR (93.9% vs. 83.1%, P=0.039, **Figure 2a**). Conversely, no statistically significant differences were observed in OS survival were observed (89.9% vs. 88.1%, P=0.296, **Figure 2b**).

**Figure 3:**
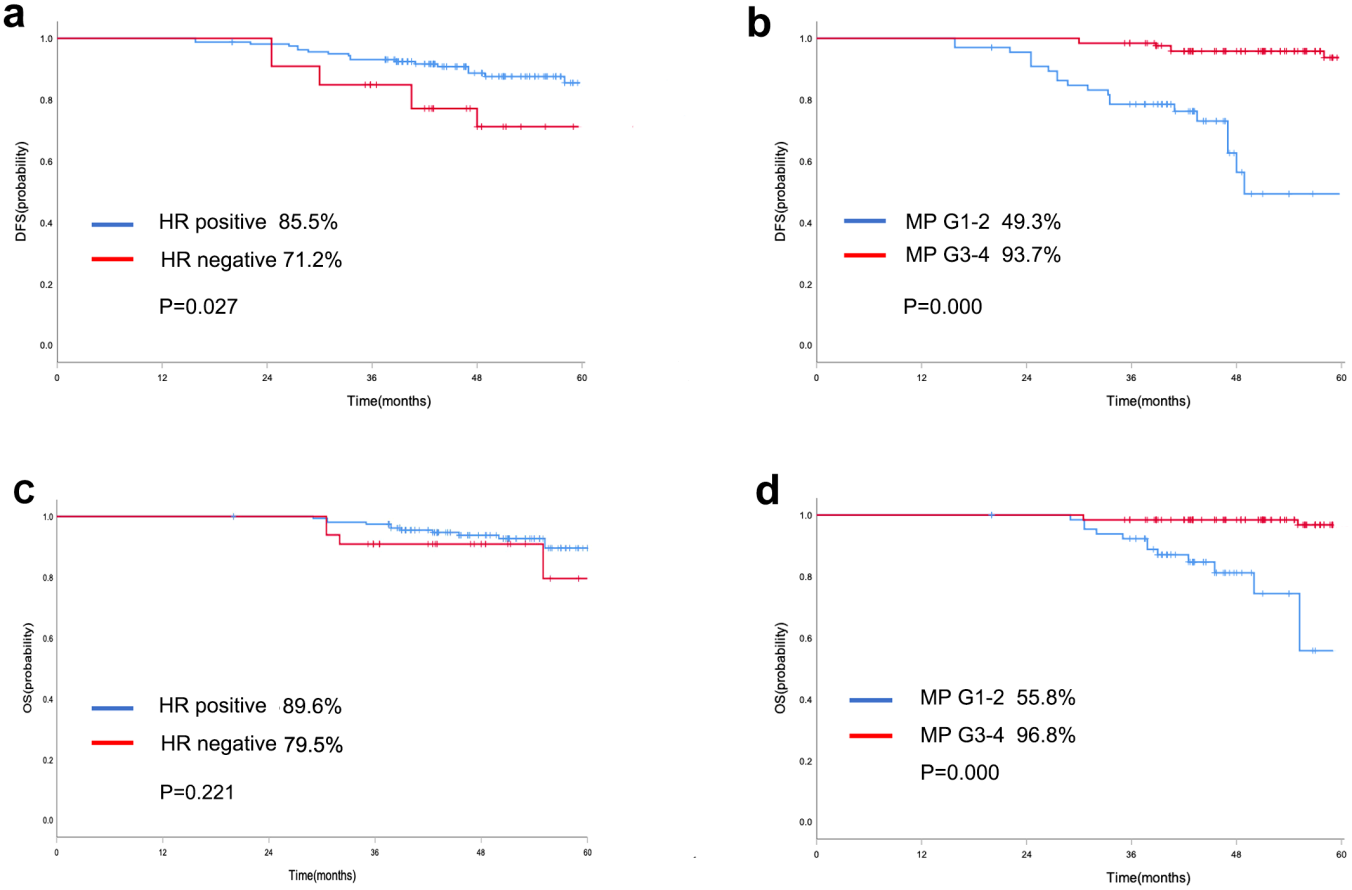
Kaplan-Meier curve of DFS in patients who did not achieve pCR with different HR status **(a)** and MP grading system **(b)**; Kaplan-Meier curve of OS in patients who did not achieve pCR with different HR status **(c)** and MP grading system **(d)**.

Survival analysis was conducted on the 194 patients who did not achieve pCR. For DFS, in univariate analysis, MP grade 3–4 (HR: 0.090; 95% CI: 0.035–0.234; p=0.000) and HR negative status(HR: 2.479; 95% CI: 1.075-5.712; p=0.033) were identified as variables associated with survival outcome among patients who did not achieve pCR (**Table 3**). Furthermore, MP grade 3–4 (HR: 0.094; 95% CI: 0.037–0.238; p=0.000) and HR negative status (HR: 2.561; 95% CI: 1.100–5.966; p=0.029) were shown to be significant independent predictors of DFS by multivariable analysis (**Figure 3**, **Supplementary Figure S1**). The multivariate analysis revealed that the sole significant independent predictor of OS was MP grade 3–4 (HR: 0.071; 95% CI: 0.019–0.260; p=0.000) (**Table 4**).

**Table 3:** Univariate and multivariable analyses of DFS in patients not achieving pCR.

Characteristic	Univariate analysis		Multivariate analysis	
	HR (95% CI)	*P*	HR (95% CI)	*P*
Age		0.589		
≤ 35	Ref			
> 35	0.797 (0.349-1.820)			
cT		0.885		
T1-2	Ref			
T3-4	1.060 (0.483-2.326)			
cN		0.513		
N0-1	Ref			
N2-3	1.287 (0.604-2.741)			
Clinical stage		0.139		
II	Ref			
III	1.867 (0.816-4.269)			
HR		**0.033**		**0.029**
Positive	Ref		Ref	
Negative	2.479 (1.075-5.712)		2.561 (1.100-5.966)	
pre Ki-67		0.785		
≤ 30%	Ref			
> 30%	1.128 (0.476-2.670)			
Surgery		0.161		
Mastectomy	Ref			
Breast conserving+IBR	0.038 (0.000-3.657)			
pT		0.361		
T0-2	Ref			
T3-4	0.393 (0.053-2.912)			
pN		0.775		
N0-1	Ref			
N2-3	1.118 (0.518-2.413)			
Pathological stage		0.297		
I-II	Ref			
III	0.771 (0.474-1.256)			
HER2		0.556		
Stable	Ref			
Loss or Gain	0.746 (0.281-1.980)			
post Ki-67		0.128		
≤ 30%	Ref			
> 30%	0.438 (0.151-1.267)			
MP		**0.000**		**0.000**
1-2	Ref		Ref	
3-4	0.090 (0.035-0.234)		0.094 (0.037-0.238)	

HR, hormone receptor; IBR, immediate breast reconstruction; MP, Miller/Payne grading system. Bold value: there are statistical differences.

**Table 4:** Univariate and multivariable analyses of OS in patients not achieving pCR.

Characteristic	Univariate analysis		Multivariate analysis	
	HR (95%CI)	*P*	HR (95%CI)	*P*
Age		0.529		
≤ 35	Ref			
> 35	1.497 (0.427-5.255)			
cT		0.069		0.087
T1-2	Ref		Ref	
T3-4	0.252 (0.057-1.112)		0.272 (0.061-1.208)	
cN		0.837		
N0-1	Ref			
N2-3	0.901 (0.335-2.422)			
Clinical stage		0.946		
II	Ref			
III	0.967 (0.360-2.599)			
HR		0.231		
Positive	Ref			
Negative	2.000 (0.643-6.228)			
pre Ki-67		0.706		
≤ 30%	Ref			
> 30%	1.243 (0.401-3.859)			
Surgery		0.274		
Mastectomy	Ref			
Breast conserving+IBR	0.038 (0.000-13.459)			
pT		0.388		
T0-2	Ref			
T3-4	0.042 (0.000-56.975)			
pN		0.978		
N0-1	Ref			
N2-3	0.986 (0.358-2.714)			
Pathological stage		0.327		
I-II	Ref			
III	0.729 (0.388-1.370)			
HER2		0.345		
Stable	Ref			
Loss or Gain	0.489 (0.111-2.160)			
post Ki-67		0.137		
≤ 30%	Ref			
> 30%	0.030 (0.000-3.047)			
MP		**0.000**		**0.000**
1-2	Ref		Ref	
3-4	0.070 (0.019-0.255)		0.071 (0.019-0.260)	

HR, hormone receptor; IBR, immediate breast reconstruction; MP, Miller/Payne grading system. Bold value: there are statistical differences.

## Discussion

With the emergence of novel ADC drugs and the release of the DESTINY-Breast 04 and the ASCENT clinical trials results, the concept of HER2-low breast cancer has attracted widespread attention (21–23). In metastatic breast cancer with low HER2 expression, the DESTINY-Breast 04 study assessed the clinical efficacy of trastuzumab deruxtecan (T-DXd) in comparison with that of conventional chemotherapy. The progression free survival (PFS) of the T-DXd group was substantially better than that of the control group (10.1 months vs. 5.4 months; HR 0.51, P < 0.001). This pivotal phase III clinical study, DESTINY-Breast 04, proved that low HER2 expression can serve as a therapeutic category for breast cancer. Do HER2-low breast cancers differ biologically from HER2-zero tumors, making them a separate molecular subtype? The biological properties of HER2-low breast cancer, which is identified by an IHC score of 1+ or 2+/ISH-, have been the subject of numerous investigations. Nevertheless, there have not been consistent findings across studies (24–26). These results suggest that breast cancers with low HER2 status constitute a physiologically diverse group of tumors.

Our previous study indicated that the proportion of HR positive patients in HER2-low breast cancer was higher than in HER2-zero breast cancer patients, which is consistent with findings from several other studies (27, 28). In HER2-low tumors, the major determinant of the gene expression profile is HR status, with the majority of HR positive tumors belonging to the luminal A or B subtype, and the majority of HR-negative tumors classified as the basal-like subtype. After adjusting for HR status, only marginal differences in gene expression between HER2-low and HER2-zero tumors were found, highlighting that these entities are not substantially different in terms of biology (29). Similarly, after adjusting for HR status, no discernible and regular variations in the genomic profiles of HER2-low and HER2-zero tumors were discovered in large-scale genomic investigations. HER2-low breast cancer should not be considered a distinct molecular subtype, but HER2-low can be used as a therapeutic target and the biological behavior of HER2-low breast cancer need further exploration (30).

In a pooled analysis of four prospective neoadjuvant clinical trials (GeparSepto, GeparOcto, GeparX, Gain-2 neoadjuvant), HER2-low breast cancer presented a significantly lower pCR rate than HER2-zero patients did (17). However, there are also studies that have yielded inconsistent results. The differences in pCR rate between HER2-low and HER2-zero patients may be associated with the definition of pCR and HR status. In the present study, 33.8% of patients achieved pCR, and the pCR rate in TNBC was significantly higher than that observed in HR positive breast cancer patients. Specifically, the pCR rate of HR-positive patients was only 27.5%; thus, optimizing the NAC regimen is challenging, and unmet clinical needs remain. ADC drugs are expected to have potential application in the use of neoadjuvant therapy to treat early HER2 positive and HER2-low breast cancer patients.

T-DXd monotherapy or T-DXd sequential THP compared with ddAC-THP is being actively studied in the clinical trial DESTINY-Breast11 (DB11) for high-risk HER2-positive early breast cancer. This novel neo-adjuvant therapy scheme is expected to further increase the pCR rate of patients and reduce the treatment burden and overall toxicity of patients. A phase II study of TALENT (TRIO-USB-12) aimed to evaluate the clinical efficacy and safety of T-DXd monotherapy or T-DXd combined with endocrine therapy in the treatment of early HR positive HER2-low breast cancer patients. By the data cut-off date, the objective response rate (ORR) of T-DXd monotherapy group was 68%(17/25), whereas that of T-DXd combined with endocrine therapy group was 58%(14/24). The results showed that the ORR of T-DXd monotherapy are equivalent to the existing data of aromatase inhibitor neoadjuvant therapy for HR+/HER2 negative breast cancer. Although the data about the pCR rate remain immature at this stage, the existing data of efficacy and safety data show that T-DXd neoadjuvant therapy is worth exploring.

Currently, few clinical studies have focused on prognostic factors for HER2-low breast cancer after NAC. In this study, we assess the predictors of outcomes in patients with HER2-low breast cancer following NAC. Better DFS is associated with achieving a pathological complete response to NAC. However, no statistically significant differences in OS were observed. pCR has been proposed as a surrogate endpoint for prediction of long‐term survival for breast cancer receiving NAC (31). However, the issue remains controversial (32–34). According to the CTNeoBC pooled analysis of NAC clinical trials, among patients with TNBC, the strongest correlation was observed between pCR and long-term outcomes(EFS: HR 0.24, 95% CI 0.18-0.33; OS: 0.16, 0.11-0.25), particularly among patients with HR-negative/HER2-positive cancers. Our present study explored the relationship between pCR and survival outcomes in HER2-low breast cancer patients. With pCR defined as ypTis/0ypN0, HER2-low breast cancer patients who achieved pCR had better DFS. Thus, pCR may be serve as a predictor for DFS in HER2-low breast cancer. The question of whether the pCR obtained after NAC can be transformed into the long-term survival benefit of the whole population of patients with breast cancer is still controversial.

Our findings show that the dichotomization of patients is a significant limitation of pCR. The response of NAC varied greatly for patients who did not achieve pCR. The vast majority of DFS events occur in patients who have not obtained pCR. Therefore, a prognostic study was carried out in patients who did not achieve pCR. The pathology evaluation system that is currently most widely utilized in China is the Miller−Payne grading system. Previous studies confirmed that MP grading system was a useful supplement and could provide more prognostic information besides pCR (35, 36). However, the prognostic value of MP grading system in HER2-low breast cancer have not yet been reported. In this study, multivariable analysis demonstrated that MP grading system was significant independent predictor of DFS and OS, indicating that pathological evaluation after NAC provide important information of survival outcome for HER2-low breast cancer. Neoadjuvant therapy allows clinicians to optimize intensive adjuvant therapy based on the patient’s response to therapy. The CREATE-X and KATHERINE studies showed that capecitabine and T-DM1 improved 5-year DFS and 3-year iDFS rates by 13.7% and 11.3%, respectively, in TNBC and HER2-positive breast cancer patients who did not achieve pCR (37, 38). Similarly, unmet clinical needs remain in HER2-low breast cancer patients who do not achieve pCR, and intensive adjuvant therapy may also be necessary.

In the context of neoadjuvant therapy, HR-positive breast cancer is less sensitive to treatment, and the PCR rate is usually lower than that of triple-negative breast cancer. However, many clinical studies have shown that a high expression of HR is related to a better prognosis. This may be attributed to the use of endocrine therapy for HR-positive breast cancer patients.

The present study does have some limitations. First, this was a retrospective study. Second, the number of cases included in this study was not very large.

Prospective clinical research is needed to verify the value of MP grading system in HER2-low breast cancer. Third, HER2 expression data was retrieved from medical records, and reviewed by the pathologist, these data were not evaluated by multiple pathologists. Nevertheless, to our knowledge our study provides information on factors that predict outcomes of HER2-low breast cancer patients after NAC for the first time.

## Conclusion

The results of our study show that pathological assessment following NAC offers valuable insights into the survival outcome of HER2-low breast cancer. On the basis of these findings, patients with HER2-low breast cancer should have their response to NAC taken into account when choosing a systemic treatment.

## Data Availability

The raw data supporting the conclusions of this article will be made available by the authors, without undue reservation.

## Supplementary material

Supplementary Figure 1 The nomogram to predict DFS of patients based on HR and MP grading system.
